# MR Thermometry Data Correlate with Pathological Response for Soft Tissue Sarcoma of the Lower Extremity in a Single Center Analysis of Prospectively Registered Patients

**DOI:** 10.3390/cancers12040959

**Published:** 2020-04-13

**Authors:** Michaela Unsoeld, Ulf Lamprecht, Frank Traub, Barbara Hermes, Marcus Scharpf, Vlatko Potkrajcic, Daniel Zips, Frank Paulsen, Franziska Eckert

**Affiliations:** 1 Department of Radiation Oncology, Eberhard-Karls-University Tuebingen, Hoppe-Seyler-Str. 3, 72076 Tuebingen, Germany; michaela.unsoeld@student.uni-tuebingen.de (M.U.); Ulf.Lamprecht@med.uni-tuebingen.de (U.L.); Vlatko.Potkrajcic@med.uni-tuebingen.de (V.P.); Daniel.Zips@med.uni-tuebingen.de (D.Z.); frank.paulsen@med.uni-tuebingen.de (F.P.); 2 Centre for Soft Tissue Sarcoma, GIST and bone tumors, Eberhard-Karls-University Tuebingen, Otfried-Mueller-Straße 10, 72076 Tuebingen, Germany; frank.traub@unimedizin-mainz.de (F.T.); Barbara.Hermes@med.uni-tuebingen.de (B.H.); Marcus.Scharpf@med.uni-tuebingen.de (M.S.); 3 Department of Orthopaedic Surgery, Eberhard-Karls-University Tuebingen, Hoppe-Seyler-Str. 3, 72076 Tuebingen, Germany; 4 Center for Orthopedic and Trauma Surgery, Johannes Gutenberg-University Mainz, Langenbeckstr. 1, 55131 Mainz, Germany; 5 Department of Medical Oncology, Eberhard-Karls-University Tuebingen, Otfried-Mueller-Str. 10, 72076 Tuebingen, Germany; 6 Institute for Pathology, Eberhard-Karls-University Tuebingen, Liebermeisterstraße 8, 72076 Tuebingen, Germany; 7 German Cancer Research Center (DKFZ), German Cancer Consortium (DKTK) Partnersite Tuebingen, Im Neuenheimer Feld 280, 69120 Heidelberg, Germany

**Keywords:** Sarcoma, radiotherapy, hyperthermia, MR thermometry, pathological response

## Abstract

*Background*: There is a strong biologic rationale for using locoregional hyperthermia in soft tissue sarcoma and a randomized trial reported significant improvements with hyperthermia. The aim of this study was to describe the opportunities of magnetic resonance (MR)-based thermometry in a cohort of soft tissue sarcoma patients undergoing combined radiotherapy and locoregional hyperthermia. *Patients and Methods*: For eleven evaluable patients, tumor volume (V_Tu_) and a separate volume for temperature analysis with reliable temperature distribution (V_therm_) were contoured for every hyperthermia treatment (103 therapies). Temperature data were recorded for all tumors and were correlated with clinical features and pathologic response data. *Results*: Of 48 patients with high-risk soft tissue sarcomas treated with radio(chemo)therapy and locoregional hyperthermia, MR thermometry was possible in 11 (23%) patients. For all patients, the temperature superseded by 90% of V_Tu_ (T90(V_Tu_)) and T90 (V_therm_) were in the range of 37–43 °C and 40–45 °C, respectively. Larger tumors tended to reach higher temperatures. For tumors showing a pathologic response in the resection specimen after preoperative treatment, temperature (T90 (V_therm_)) was significantly higher than in tumors without pathologic response. *Conclusion*: Lower extremity sarcomas undergoing preoperative treatment with locoregional hyperthermia are especially suitable for MR thermometry. MR thermometry is a promising non-invasive way for temperature measurement during locoregional hyperthermia, showing a positive dose-response relationship.

## 1. Introduction

Locoregional hyperthermia has been established for the treatment of adult-type soft tissue sarcoma in the neoadjuvant and adjuvant settings as an addition to chemotherapy and/or radiotherapy [1]. A landmark randomized clinical trial compared neoadjuvant or adjuvant chemotherapy +/− radiotherapy alone versus the same therapy complemented by locoregional or partial body hyperthermia twice weekly. In 2010, a significantly prolonged disease-free survival for the hyperthermia treatment arm was reported [2]. The positive results were confirmed with a significantly improved disease-specific survival in 2018 [3]. The biologic rationale for additive/synergistic effects of locoregional hyperthermia combined with radiotherapy are multidimensional including better tumor perfusion, inhibited repair of DNA damages and probably immune-mediated effects [4].

With oncologic outcomes being comparable for pre- and postoperative radiotherapy [5] and better long-term functional outcome for preoperative radiation [6,7], neoadjuvant treatment has become a standard approach at several institutions, even taking into account enhanced wound complications [5]. Our institutional standard follows the IAWS protocol (Interdisziplinaere Arbeitsgruppe Weichgewebesarkome) [8], adding ifosfamide chemotherapy and locoregional hyperthermia to radiotherapy simultaneously. This strategy is successful even for macroscopic sarcomas without complete resection [9]. While local recurrences of soft tissue sarcoma after surgery alone had a significantly impaired local control rate, multimodal treatment is able to achieve local tumor control in over half of the patients [10]. In a retrospective analysis, multimodal treatment led to superior disease-specific survival compared to radiotherapy alone [11]. This treatment strategy has been described by several sarcoma centers [12,13,14,15,16,17]. For highest risk sarcomas (deep-seated, large, grade 3) in younger patients, 4–6 additional cycles of doxorubicin and ifosfamide chemotherapy can be offered [18].

Temperature measurement during hyperthermia treatment of sarcomas can be done either by direct thermometry using invasive thermometry probes or by indirect methods like magnetic resonance (MR)-based proton resonance frequency shift images indicating temperature change over time [19]. Taking into account known pitfalls, MR thermometry adds to interstitial probes in several aspects. Spatial temperature distribution can be visualized, detecting cold spots in the tumor as well as hot spots in normal tissue. For deep seated tumors in particular, in which interstitial probe thermometry might be difficult to achieve, MR-based thermometry enables non-invasive temperature surveillance [20]. Several groups have done comparison studies of MR-based thermometry and interstitial temperature measurements showing them comparable [21], especially in large soft tissue sarcomas. Craciunescu et al. described that the differences between invasive and MR-based measurements (in small volumes of interest close to the interstitial probes) were below 1 °C [22]. Gellermann et al. confirmed these results and established a correlation of temperature data with the pathologic response of tumors treated neoadjuvantly [23].

With this background, the aim of this study was to identify subgroups of patients treated with radiotherapy and hyperthermia (+/− chemotherapy) for high risk, adult type soft tissue sarcoma, in which MR-guided thermometry is feasible. Thermometry was done in the whole tumor volume (V_Tu_) as well as a subvolume of the tumor (V_therm_) using T90 (minimal temperature in 90% of the contoured volume) and CEM43 (cumulative equivalent minutes of exposure at 43 °C). The temperature characteristics were evaluated over the course of treatment and correlated with clinical features such as tumor size and pathologic response.

## 2. Results 

### 2.1. Patient Selection and Patient Characteristics

**Figure 1:**
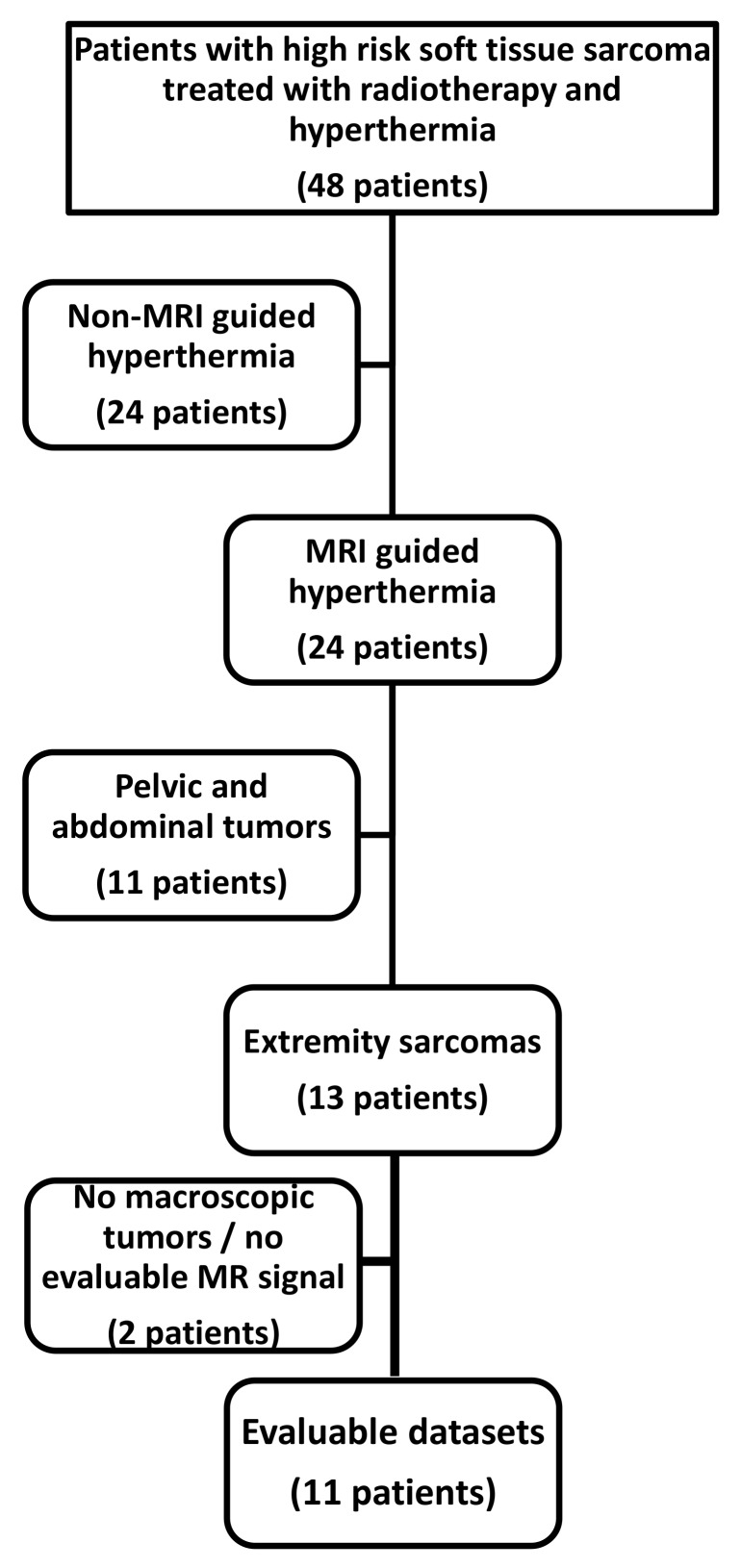
Of 48 patients registered in the prospective database of our institution of soft tissue sarcoma patients treated with radiotherapy and locoregional hyperthermia ± chemotherapy, 11 datasets were identified with neoadjuvant treatment of lower extremity tumors treated with MR-guided hyperthermia, which then were used for the analysis.

Of 48 patients treated with combined radiotherapy and locoregional hyperthermia (with or without sequential or concomitant chemotherapy), 11 (23%) met the inclusion criteria for this study (MR-guided hyperthermia of lower extremity sarcomas with neoadjuvant treatment intent, Figure 1).

Patients treated with non-MR guided hyperthermia include patients with sarcomas of the upper extremities which cannot be treated in the combined MR-hyperthermia unit. Other patients treated with regional hyperthermia are patients requiring treatment in the Sigma60 applicator not compatible with the MR (due to patient diameter) or patients with general MR contraindications such as severe hearing loss or pacemakers. 

Retroperitoneal, abdominal and pelvic tumors were excluded, as breathing and intestinal motion disturbs MR-based thermometry. All patients had large, subfascial tumors, grade two or three (Fédération Nationale de Centres de Lutte Contre le Cancer, FNCLCC), and thus were categorized as high-risk soft tissue sarcomas. Treatment was performed in a neoadjuvant setting enabling intratumoral MR-based thermometry during the course of treatment. Six patients received trimodal therapy including two cycles of ifosfamide chemotherapy concomitant to radiotherapy. Details are given in Table 1.

### 2.2. Contouring and Quality Assurance

**Figure 2:**
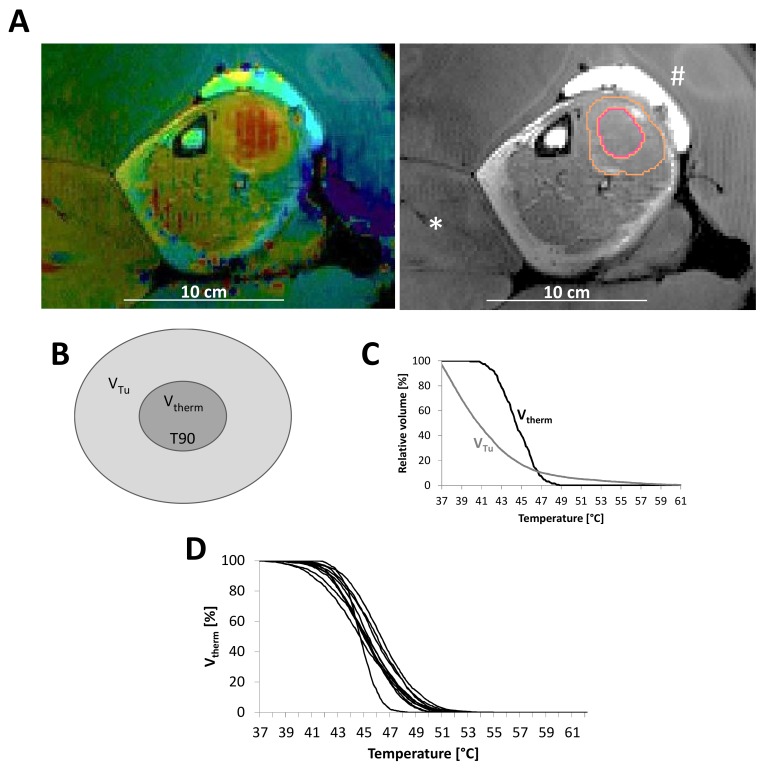
An example of a sarcoma of the calf shows the use of water bags (*) between the legs, as well as oil bags (#) to enhance the temperature in the tumor close to the skin surface. The outer contour (orange) depicts the tumor volume, the inner contour (red) indicates the volume used for thermometry (V_therm_) (**A**) as also indicated in the schematic view (**B**). Comparing temperature-volume histograms for the whole tumor volume (V_Tu_) versus V_therm_ show a more reliable temperature distribution for V_therm_ (**C**). Temperature-volume histograms for all treatments of one patient show a quite consistent temperature distribution and small intrapatient variability (**D**).

Contoured tumor volumes in T2 weighted imaging for hyperthermia correlated well with gross tumor volume (GTV) contoured on radiotherapy planning computed tomography (CT)s (co-registered to pre-therapy diagnostic MR images) with a Pearson correlation coefficient of 0.92. Additional thermometry volumes contoured for every timepoint of hyperthermia treatment in areas of reliable temperature signal (V_therm_) accounted for mean 21.9 ± 1.5% of the entire tumor volume (V_Tu_). For several patients, temperature-volume histograms for V_Tu_ as well as V_therm_ showed reliable data. However, as shown in Figure 2, for some tumors the data for V_Tu_ were unreliable with temperatures reaching from 38 to 57 °C.

Quality assurance consisted of creating voxel-based temperature-volume histograms of the proton resonance frequency shift datasets. Datasets with motion artifacts showed negative slopes at 36 °C or lower (observed for pelvic and abdominal tumors). However, datasets used for the evaluation showed smooth temperature-volume histograms with a fall-off starting at >38 °C for the treatment time (Figure 2). T90 was extracted from every temperature-volume histogram manually, means were calculated for every single hyperthermia treatment as well as for all hyperthermia treatments of one patient. Mean T90 (V_therm_) for all treatments of all patients was 43.0 ± 0.21 °C. Mean calculated CEM43 (V_therm_) for all treatments of all patients was 3.7 ± 0.73 h. Mean total CEM43 (V_therm_) per patient (summed up over all hyperthermia treatments for one patient) was 35.9 ± 18.1 h.

### 2.3. T90 and CEM43 during Course of Therapy

**Figure 3:**
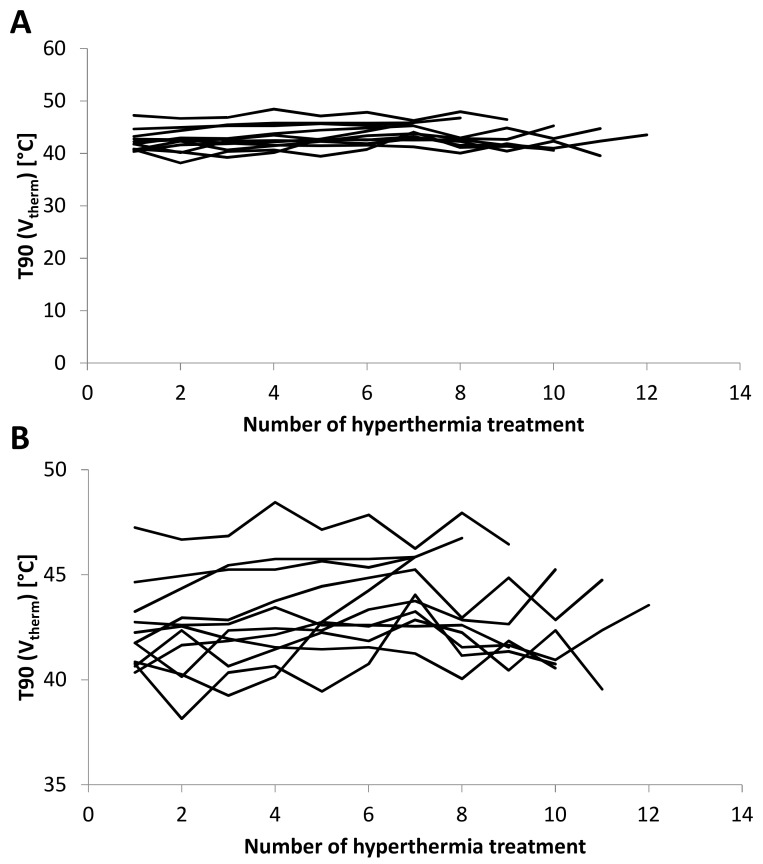
T90 (V_therm_) is shown for every patient over the course of radiotherapy for every hyperthermia treatment. T90 (V_therm_) was in the range of 38 °C to 46 °C for all patients. Stable temperature for hyperthermia treatments over the course of radiotherapy indicates a good tolerability. All patients received 7 or more hyperthermia treatments (**A**). The lower panel shows a smaller temperature range to indicate the inter- and intra-individual differences (**B**).

In most patients, T90 (V_therm_) and CEM43 (V_therm_) were rather stable over hyperthermia treatments during radiotherapy. Interpatient variations were larger than intrapatient variations in between treatments. Of 104 treatments, 100 showed a T90 (V_therm_) of 40 °C or more (range: 38.2–48.5 °C). Due to the mathematical equation for CEM43 and temperatures in the range of 38 °C to 48 °C CEM43 (V_therm_) showed much more variation compared to T90 (V_therm_). No patient discontinued therapy. A tendency towards increased T90 (V_therm_) and CEM43 (V_therm_) over the weeks of radiotherapy points toward good tolerability of hyperthermia concurrent with radiotherapy. Hyperthermia treatments for all patients are shown in Figure 3.

### 2.4. Correlation of Mean T90 and Cumulative CEM with Tumor Size

**Figure 4:**
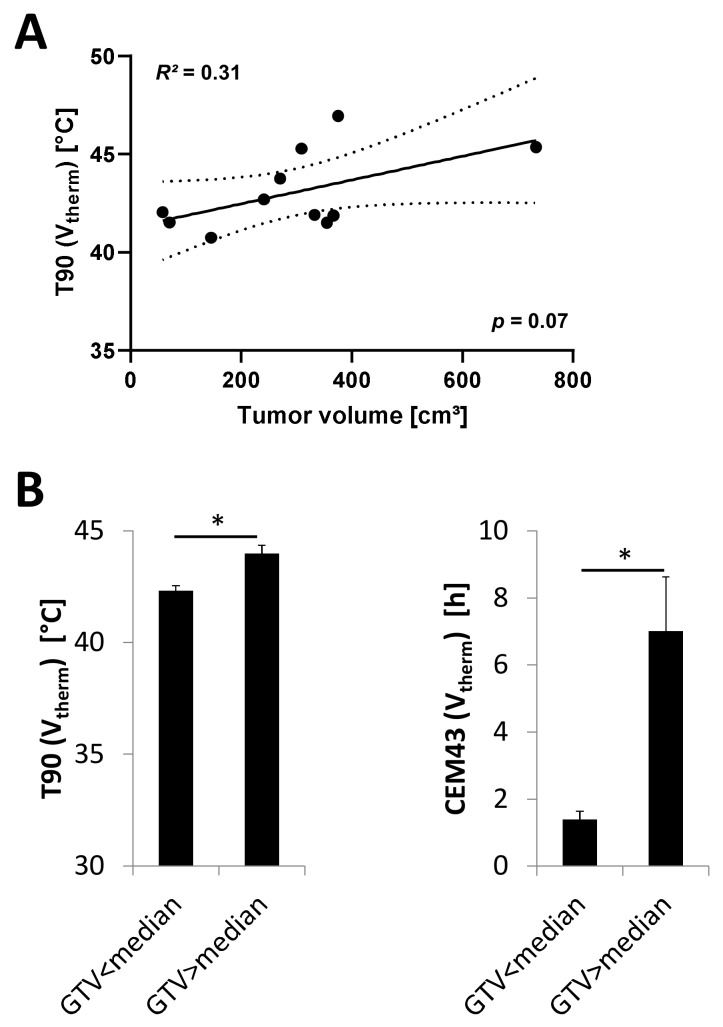
Mean T90 (V_therm_) for all timepoints of all hyperthermia treatments for individual patients showed a moderate correlation with a trend to statistical significance with the contoured tumor volume. Large tumors tended to reach higher temperatures (**A**). T90 (V_therm_) as well as CEM43 (V_therm_) was significantly higher for tumors with GTV volume above the median compared to tumors with GTV size below the median (**B**). * *p* < 0.05

Tumors were large with a mean diameter of 10.4 ± 1.0 cm. All tumors had a diameter larger than 5 cm. Mean GTV (derived from the radiation treatment plans) was 354.2 ± 65.9 cm^3^. Linear regression of mean T90 and contoured tumor volume showed a positive correlation with a trend to statistical significance (*R^2^* = 0.31, *p* = 0.07, Figure 4). With a cut-off of 348.8 cm^3^ (median GTV size), large tumors reached a significantly higher mean T90 (V_therm_). The sum of CEM43 (V_therm_) for all treatments per patient was also significantly longer for tumors with a GTV > 348.8 cm^3^ (Figure 4). 

### 2.5. Correlation of Temperature with Pathologic Response

**Figure 5:**
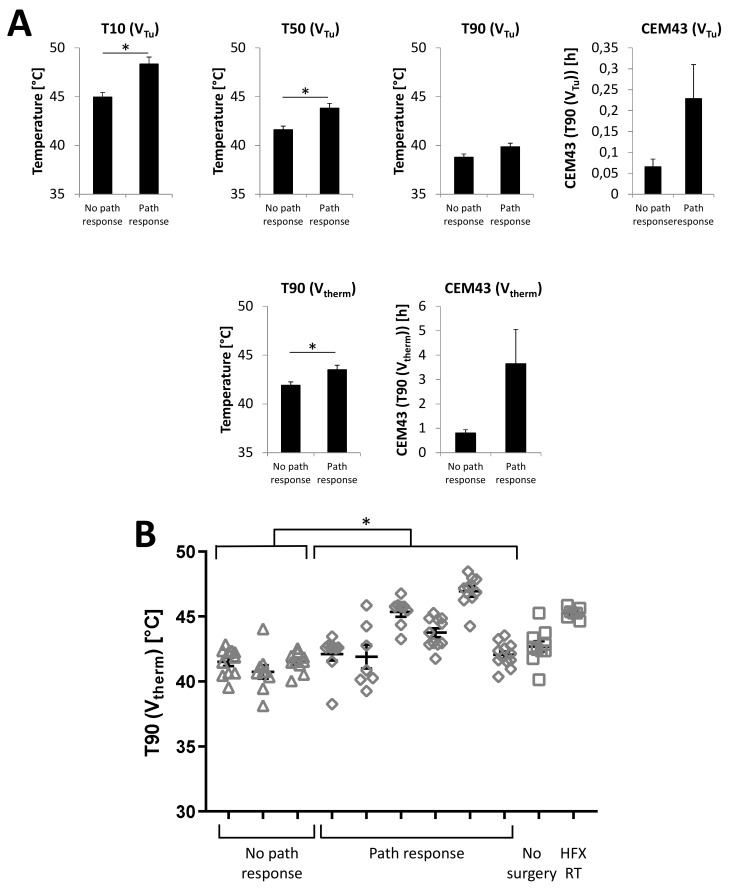
Mean T10 (V_Tu_) and T50 (V_Tu_) as well as mean T90 (V_therm_) were significantly higher in patients who achieved a pathologic response with <10% vital tumor cells in the resection specimen (three hyperthermia sessions per patient) (**A**). T90 (V_therm_) for every hyperthermia treatment grouped for individual patients shows lower temperatures for patients with >10% vital tumor cells in the resection specimens (triangles) compared to patients achieving a pathologic response (rhombuses). Excluded are the patient not undergoing surgery and the patient who had received hyperfractionated radiotherapy (squares) (**B**). * *p* < 0.05

Pathologic response was not assessable in one patient, as no surgery was performed due to the detection of metastatic disease in pre-resection imaging. Another patient was excluded from the analysis as he had received hyperfractionated radiotherapy and pathologic response is not comparable to the other patients due to the different time course of hyperthermic radiotherapy and surgery. T90 (V_therm_) showed a moderate negative correlation with remaining vital tumor cells in the surgical specimen after neoadjuvant therapy (Pearson correlation coefficient of −0.51. The evaluation of temperature data from three hyperthermia sessions for each evaluable patients showed significant correlations with pathologic remission of T10 (V_Tu_), T50 (V_Tu_) and T90 (V_therm_), but not for T90 (V_Tu_), CEM43 (V_Tu_) and CEM 43 (V_therm_), as shown in Figure 5. Single values of T90 (V_therm_) from hyperthermia treatments of every patient show low T90 (V_therm_) values throughout all hyperthermia treatments for all three patients without a pathological response, whereas patients with a pathological response showed large interpatient variability for T90 (V_therm_) (Figure 5). Overall, T90 (V_therm_) was significantly higher for those patients.

Pathologic response was independent from initial tumor volume as indicated by mean tumor volumes for patients with and without a pathological response of 356.1 ± 89.3 cm^3^ and 218.8 ± 67.1 cm^3^, respectively (*p* = 0.30). Change of tumor volume did not differ significantly between patients with pathologic response (relative tumor volume 0.88 ± 0.12) and without pathologic response (relative tumor volume 0.96 ± 0.07) with *p* = 0.72. Sequential and simultaneous chemotherapy did not influence the pathologic response rate.

## 3. Discussion 

Our study identifies large, high-risk soft tissue sarcomas of the lower extremities during neoadjuvant therapy as especially suited for partial body hyperthermia with MR-guided thermometry during neoadjuvant therapy. Large tumors facilitate the contouring of thermometry volumes with reliable temperature signal covering significant portions of the tumor volume in most cases. In lower extremity tumors, hyperthermia in the combined MR-hyperthermia unit is possible in contrast to upper extremity sarcomas. In addition, compared to pelvic and abdominal sarcomas, breathing and intestine motion artifacts do not play a role in this anatomical region. With these criteria, evaluation of MR thermometry was possible in 23% of all sarcoma patients treated with a radiotherapy and hyperthermia combination at our institution. T90 (V_therm_) for all patients, corresponding to T50 of the whole tumor volume, was 43.0 °C and thus in the range of previously published datasets [23]. MR-based thermometry enables non-invasive temperature monitoring in sarcomas not accessible to direct temperature probes without placing them in the tumor (in contrast to rectal or bladder cancer with direct access to the tumor region by temperature probes). Thus, MR thermometry is a very useful method to be able to correlate temperature data with clinical outcome in sarcoma patients.

To our knowledge, this is the first dataset establishing a relationship between tumor size and temperature reached during locoregional hyperthermia treatment. A possible explanation might be better tolerability of high temperature in central tumor regions of large sarcomas. Good temperature tolerability might also explain the higher temperatures reached in our sarcoma patient cohort compared to pelvic malignancies (in close proximity to mucosa and skin highly sensitive to pain) [24]. Locoregional hyperthermia has been described to lead to higher rates of pathological response in rectal cancer, especially in patients receiving at least four hyperthermia treatments [25]. For sarcomas, our dataset as well as the data reported by Gellermann et al. [23] establish a dose-response relationship of temperature and pathological response. For patients with pathological remission, higher T90 and CEM43 were reported, which is confirmed by our results. In concordance with previously published data, the two patients in our cohort with molecularly confirmed myxoid liposarcomas showed a pathologic response [26].

Mechanisms discussed for the positive effect of hyperthermia on pathological response and oncologic outcome in combination with radiotherapy and/or chemotherapy include cellular changes leading to higher radio- and chemosensitivity, changes in tumor perfusion counteracting hypoxic microenvironments and immune mechanisms of enhanced anti-tumor immune responses. Synergistic effects relating to cell death and antiproliferative effects have been described for several carcinoma cell lines in vitro [27,28,29,30]. The same was found for sarcomas [31]. DNA-double-strand-break-repair inhibition has been identified as an intracellular mechanism of enhanced radiosensitivity in combination with hyperthermia [32].

Tumor perfusion was significantly enhanced during locoregional hyperthermia of pelvic tumors as measured by direct probes as well as perfusion measured by positron-emission-tomography (PET) [33,34]. This leads to improved oxygenation of sarcomas during radiotherapy and hyperthermia and thus, indirectly, to increased radiosensitivity [35]. A recent focus in hyperthermia research is immune modulation [36]. Heat shock proteins are potent activators of the immune system [37,38,39]. The supernatant of tumor cells treated with radiotherapy and hyperthermia induced stronger transwell migration of immune cells compared to control supernatant [40]. Hyperthermia enhances maturation of dendritic cells [41,42] and might be able to reprogram immune-suppressive regulatory T-cells [43]. Thus, the correlation of temperature values and pathologic response (establishing a dose-response-relationship for locoregional hyperthermia in soft tissue sarcoma) is based on a strong biological rationale.

## 4. Patients and Methods

Radiotherapy was scheduled according to institutional standards. All but one patient were treated with normofractionated neoadjuvant radiotherapy (1.8–2.0 Gy/fraction) to a total dose of 50.0–50.4 Gy (total treatment time 5–6 weeks, surgery after 5–6 weeks). One patient was treated with hyperfractionated radiotherapy due to fast tumor progression before the start of therapy (1.6 Gy/fraction, twice daily) to a total dose of 48.0 Gy (total treatment time 3 weeks, surgery after 3 weeks). In six patients two cycles of ifosfamide (3.0 g/m^2^ body surface area, d1 + 2) were administered concurrently. Five patients had received 4 cycles of neoadjuvant chemotherapy with doxorubicin / ifosfamide before start of radiotherapy. 

MR thermometry data of good quality were available for 103 therapies in 11 patients (median 10 hyperthermia treatments per patient) with high risk adult type soft tissue sarcoma of the lower extremity treated neoadjuvantly with combined radiotherapy and partial body hyperthermia using an integrated MR-hyperthermia unit BSD 2000/3D MR (Pyrexar Medical, formerly BSD medical corporation, Salt Lake City, UT, USA) [44]. Treatment was planned with SigmaHyperPlan^®^ V2.01 (Dr. Sennewald Medizintechnik GmbH, Munich, Germany). The applicators used for different anatomical regions were Sigma 30 and Sigma Eye. MR-temperature control every 10 min with SigmaVision^®^ V1.01 (part of SigmaHyperPlan^®^) was used for online adaptation of steering parameters. Hyperthermia was performed twice weekly during the course of radiotherapy. Patients with retroperitoneal tumors treated with MR-guided hyperthermia were excluded due to motion artifacts in the MR thermometry datasets. Patients treated after initial surgery or at presentation with recurring tumors after previous radiotherapy were not included in the analysis due to the lack of information on pathological response and different biology and radiotherapy schedules. Patients presenting with distant metastases initially were excluded, while one patient who was treated neoadjuvantly and had developed lung metastases before the planned wide excision was included, despite lacking information on pathological response. The study was approved by the Ethics Committee of the Medical Faculty Tuebingen, with protocol code 144/2020BO2. Informed consent is not required for retrospective analysis of patient data.

At the baseline of every hyperthermia treatment, T2 weighted anatomical imaging of the tumor regions were acquired as well as proton resonance frequency shift images. Thermometry was repeated every ten minutes through the whole treatment time of 90 min (30 min heating phase, 60 min treatment phase). For every time step of every hyperthermia treatment (median 10, range 7–12/patient) the total tumor volume (V_Tu_) was contoured on T2 anatomical images. Thermometry heatmaps were co-registered. After co-registration with information from both imaging datasets a separate volume for thermometry (V_therm_) was contoured. The volume was chosen in a way that temperature-volume histograms showed reliable data starting at 36–38 °C and not reaching higher than 50 °C, and thus encompasses different percentages of the tumor volume reaching from 3% to 52%. As there are thermometry artifacts mostly at the fringe of the tumor, the volume V_therm_ mostly refers to central subvolumes of the tumor with higher temperatures. Temperature change from baseline was reported for every voxel, temperature was calculated as a change from baseline added to 36.8 °C, the temperature reported for healthy muscle of lower extremities [45]. Voxel based analysis of temperature data in the volume V_therm_ was performed as cumulative histograms. The temperature superseded by 90% of voxels of V_therm_ was defined as T90 (V_therm_). We opted for T90 even for a subvolume of the tumor as the recommendation for reporting on radiation dose or temperature for volumes with a heterogenous distribution is more reliable for “near minimal doses”, as discussed for intensity modulated radiation planning [46]. Likewise, T10 and T50 are defined as the temperature reached in 10% and 50% of the volume, respectively. Cumulative equivalent minutes at 43 °C (CEM43) was calculated as published previously [47] and analyzed as cumulative time over all treatments for individual patients. CEM43 of hyperthermia treatments which were performed but for which no thermometry data were available were substituted by the mean CEM43 of all treatments with available data.

Clinical factors taken into account for analysis were tumor volume as well as pathologic response, defined as <10% vital tumor cells in the resection specimen after neoadjuvant treatment as well as diagnosis of local or distant tumor recurrence. For the analysis of pathologic remission in relation to temperature data, three hyperthermia treatments of all patients evaluable for pathologic remission were analyzed. T10, T50 and T90 for V_Tu_, as well as T90 for V_therm_ and CEM43 (T90) for both volumes were reported. For the analysis of T90 (V_therm_) and CEM43 (V_therm_) all available datasets were used.

The statistical analysis was performed with the software package SPSS 24 (SPSS Inc., Chicago, IL, USA) and GraphPad Prism8 (GraphPad Software, San Diego, CA, USA). Means are indicated ± standard error of the mean and were compared by student’s *t*-test. Continuous variables were compared using a linear regression model and Pearson’s correlation coefficients. Crosstabs were analyzed with Chi-square test. Statistical significance was defined for a *p*-value < 0.05, a trend to statistical significance for *p*-values between 0.05 and 0.10.

## 5. Conclusions

Especially in the neoadjuvant treatment of large sarcomas of the lower limb, MR-based thermometry offers a helpful tool to acquire temperature data during locoregional hyperthermia. Thus, correlation of temperature data with clinical features and outcome becomes feasible. We found higher temperatures in larger tumors. Achieving a pathological response was associated with higher temperatures indicating a dose-response relationship for locoregional hyperthermia.

## Figures and Tables

**Table 1:** Summary of patients.

Age at Diagnosis (years)	Localisation	Side	T Stage at Diagnosis	Size (cm)	Histologic Subtype	Histologic Grading (FNCLCC)	Concomitant Ifosfamide	T90 (V_therm_) (°C)	V_therm_/V_Tu_	Path Response (% Viable Tumor)	Status at Last follow Up
36	Calf	right	2b	7.2	synovial sarcoma	2	Yes	41.5	0.26	40%	Local recurrence and distant metastases
65	Calf	right	2b	9	MPNST	3	Yes	40.7	0.08	n.a.	Distant metastases
60	Thigh	left	2b	8	sarcoma with myogenic differentiation	3	Yes	42.7	0.26	30%	Distant metastases
70	Thigh	right	2b	6.6	leiomyosarcoma	3	Yes	45.3	0.08	2%	Distant metastases
55	Thigh	left	2b	11	NOS	3	Yes	41.9	0.03	0%	NED
68	Thigh	left	2b	11	NOS	3	Yes	41.9	0.10	40%	NED
61	Thigh	left	2b	9.3	myxoid sarcoma	2	No	45.4	0.52	0%	NED
56	Thigh	right	2b	12	NOS	3	No	43.8	0.20	0%	NED
73	Thigh	right	2b	14	NOS	3	No	41.5	0.12	20%	NED
61	Calf	left	2b	17.8	myxoid liposarcoma	2	No	46.9	0.33	<5%	Distant metastases
46	Thigh	right	2b	8	myxoid liposarcoma	2	No	42.0	0.32	0%	NED

FNCLCC—Fédération Nationale de Centres de Lutte Contre le Cancer; MPNST—malignant peripheral nerve sheath tumor; NED—no evidence of disease; OS—not otherwise specified
